# New nontoxic double information magnetic and fluorescent MRI agent

**DOI:** 10.1186/2197-7364-2-S1-A88

**Published:** 2015-05-18

**Authors:** Augustinas Kublickas, Loreta Rastenien, Laima Bloznelytė-Plėšnienė, Nerijus Karalius, Marius Franckevinius, George Loudos, Amir Fahmi, Rimas Vaisnoras

**Affiliations:** Liquid Crystals Laboratory, Institute of Science and Technology, Lithuanian University of Educational Sciences, Lithuania; Institute of Physics, Center for Physical Sciences and Technology, Lithuania; Technological Educational Institute of Athens, Greece; Materials Science, Rhein-Waal University of Applied Sciences, Germany

Today sensitivity of the MRI is not enough compared to the nuclear methods, such as positron emission tomography and single photon emission computed tomography. Challenging its extension to the nanometre scale could provide a powerful new tool for the nanosciences and nanomedicine. To achieve this potential, innovative new detection strategies are required to overcome the severe sensitivity limitations of conventional inductive detection techniques. In this regard, we perform embodiment of nanodiamonds in dendrimer matrix as additional fluorescent optical and magnetic (together with Gd (III)) imaging modalities of the MRI. New hybrid system composed of dendrimer-gadolinium Gd (III) - nanodiamond as a new contrast agent for MRI was studied. Poly(propilene-imine) PPI and poly(amidoamine) PAMAM dendrimers with fixed size of nanocavities will be used as host material to protect organism against the toxicity and also to increase relaxivity of contrast agent (resulting in the increases MRI resolution). Nanodiamond as biocompatible platform to functionalize the contrast agent will be used. This bimodal hybrid system enables to use smaller amount of the contrast agent and could permit the decrease of the lateral toxicity. This bimodal hybrid system as MRI agent is providing double information (magnetic and fluorescent) about the damaged cell.

## Aim

investigation of new hybrid system composed of dendrimer-gadolinium Gd(III)-nanodiamond as a new contrast agent to improve the MRI resolution and to reduce lateral toxicity in comparison with today used. The main idea is to use this bimodal contrast agent allowing more efficient performance of the MRI with attractive biological applications in diagnosis and therapy. In this report structural investigations by SEM and TEM, spectroscopic studies by Raman scattering, absorption, fluorescence and magnetic features by EPR measurements are discussed. Obtained results are demonstrating great potential of this bimodal imaging agent with unique magnetic and optical imaging capabilities.

